# Measles virus induces persistent infection by autoregulation of viral replication

**DOI:** 10.1038/srep37163

**Published:** 2016-11-24

**Authors:** Tomomitsu Doi, Hyun-Jeong Kwon, Tomoyuki Honda, Hiroki Sato, Misako Yoneda, Chieko Kai

**Affiliations:** 1 Laboratory Animal Research Center and International Research Center for Infectious Diseases, Institute of Medical Science, The University of Tokyo, Tokyo, Japan

## Abstract

Natural infection with measles virus (MV) establishes lifelong immunity. Persistent infection with MV is likely involved in this phenomenon, as non-replicating protein antigens never induce such long-term immunity. Although MV establishes stable persistent infection *in vitro* and possibly *in vivo*, the mechanism by which this occurs is largely unknown. Here, we demonstrate that MV changes the infection mode from lytic to non-lytic and evades the innate immune response to establish persistent infection without viral genome mutation. We found that, in the persistent phase, the viral RNA level declined with the termination of interferon production and cell death. Our analysis of viral protein dynamics shows that during the establishment of persistent infection, the nucleoprotein level was sustained while the phosphoprotein and large protein levels declined. The ectopic expression of nucleoprotein suppressed viral replication, indicating that viral replication is self-regulated by nucleoprotein accumulation during persistent infection. The persistently infected cells were able to produce interferon in response to poly I:C stimulation, suggesting that MV does not interfere with host interferon responses in persistent infection. Our results may provide mechanistic insight into the persistent infection of this cytopathic RNA virus that induces lifelong immunity.

Measles is a highly contagious human disease caused by acute infection with measles virus (MV) and remains an important cause of child morbidity and mortality worldwide^1^. However, unlike most other viral diseases, infected individuals acquire life-long protective immunity against MV after recovery^2^,^3^. Although a full explanation for life-long immunity against MV has remained elusive, the 1846 island studies conducted by Panum in the Faroes and the more recent work conducted in the United States^2^, which both demonstrated life-long immunity after MV infection in the absence of reinfection, suggest the involvement of persistent MV infection in this phenomenon.

MV is a non-segmented, negative-sense, single-stranded RNA virus of the genus *Morbillivirus* within the family *Paramyxoviridae*, order *Mononegavirales*^4^. MV has six structural proteins and two accessory proteins encoded in a 16-kb RNA genome. The viral genome and replicated positive-sense viral RNA (vRNA) are encapsidated with nucleoprotein (N), making them a target of viral replication. A polymerase complex composed of phosphoprotein (P) and large protein (L) transcribes both viral mRNA and the viral genome from the encapsidated vRNA. The hemagglutinin (H) and fusion (F) proteins are involved in attachment and entry into target cells and also in syncytium formation and cytolysis. Matrix protein (M) is involved in virion budding from the infected cell. The two accessory proteins, V and C, are involved in interference with host immunity^5^.

MV is highly cytopathic and commonly causes acute severe infection, resulting in measles and, for one out of 1,000 measles cases, acute demyelinating encephalitis. However, MV also causes subacute sclerosing panencephalitis (SSPE) at a rate of one out of 1,700–3,300 measles cases if the subject is younger than five years of age when he/she is infected with MV^6^. Measles inclusion body encephalitis (MIBE) is an additional central nervous system infection that occurs in immunocompromised patients^7^. Thus, MV has the potential to cause persistent infection, although the occurrence of these diseases is very low and MV persistence has only been found in the brain in these cases. It seems likely that these diseases are induced after the long-term persistence of MV in the central nervous system; however, it cannot be excluded that MV persists elsewhere in the body. Furthermore, the persistent infection with MV in SSPE has been assumed to be a result of the appearance of mutant viruses, rather than being a natural property of wildtype strains, because many mutations have been found in the MV genomes isolated from SSPE patients^8^,^9^,^10^,^11^.

MV is known to establish persistent infection in culture^12^,^13^,^14^. The persistently infected cells show characteristic features, such as a disappearance of the cytopathic effect, a marked reduction in cell-free virion production, and a resistance to superinfection by closely related viruses. Although common mutations responsible for persistent infection have not been determined, temperature-sensitive mutants and defective interfering (DI) particles were suggested to play a role in persistent infection through restricting viral replication^15^,^16^,^17^,^18^,^19^. Interferon (IFN) was also suggested to be involved in persistent infection with MV, through restricting viral replication^20^. Receptor downregulation^21^ and receptor redistribution^22^ may account for the disappearance of syncytium formation and the resistance to superinfection with the parent virus, but this downregulation or redistribution does not account for the resistance to superinfection with closely related canine distemper virus, which does not use the same receptor, and the reduction of cell-free virion release^13^. Despite the proposition of several possibilities, however, the mechanisms responsible for MV persistent infection are still under debate.

Griffin and colleagues provided the first evidence for more reproducible persistent MV infection with MV lacking viral mutations in human and primates^23^,^24^,^25^,^26^,^27^. Additionally, MV strains isolated from the brain that are very similar to the epidemic MV strain were also reported in MIBE patients, probably due to the early onset of MIBE after a short period of persistence in immunocompromised patients^28^ These reports suggest that persistent infection is a natural property of wildtype MV. In this study, we show that MV establishes persistent infection without mutations in culture, and we unveil one potential mechanism of persistent infection by this highly cytopathic RNA virus.

## Results

### MV establishes a persistent infection in culture

**Figure 1:**
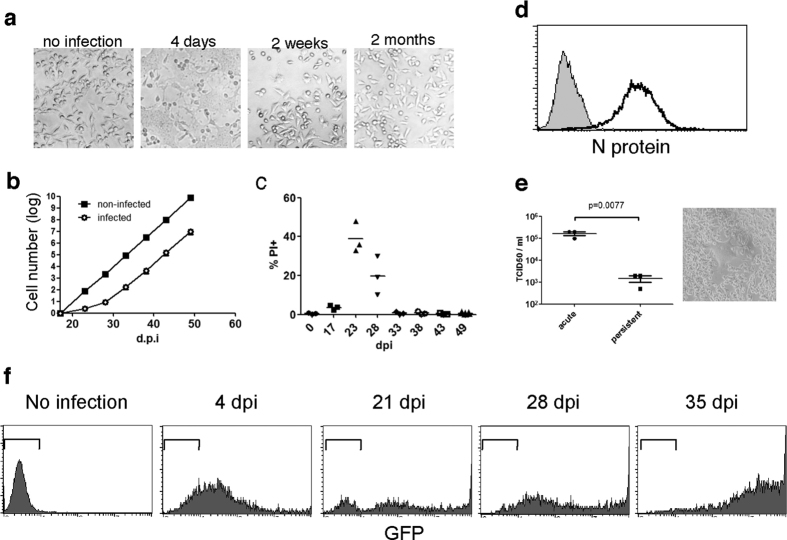
Establishment of cell lines with persistent MV infection. B95a cells were infected with rMV in (**a**–**e)**. (**a**) Cellular images of B95a cells, uninfected or at 4 days, 2 weeks, or 2 months post-MV infection. (**b**) Growth curve of B95a cells during the establishment phase of MV persistent infection. MV-infected B95a cells were counted every 5 days from 17 dpi. Control uninfected cells were also counted from same day. Error bars are SD of three independent infections. (**c**) Cell death of MV-infected B95a cells. Genomic DNA content was analysed by propidium iodide staining. The percentages of sub-G1 populations are graphed as dead cells. (**d**) Intracellular MV N protein staining of persistently MV-infected B95a cells. Filled and open histograms indicate uninfected and persistently MV-infected B95a cells, respectively. (**e**) Cell-free virion release from acute and persistent MV infection. For acute infection, B95a cells were infected with rMV-HL for 1 h at a MOI of 0.1 followed by culture medium exchange. Concurrently, equal numbers of persistently rMV-infected B95a cells were cultured, and 3 days later, the titres of three independent acute infections and three independently established persistently MV-infected cells were determined and graphed. Horizontal bars indicate averages. The cellular image shows the level of syncytia formation induced by virus released from persistently MV-infected cells. (**f**) B95a cells were infected with rMV-EGFP as described above. EGFP fluorescence was analysed by flow cytometry at the indicated times post-infection. Markers indicate the range of the EGFP-negative population.

To determine whether or not mutations in the viral genome are dispensable for the establishment of persistent MV infection, we analysed cells and viruses during the early phase of persistent MV infection in cultured cells. According to previous studies, MV establishes persistent infection in virtually any cell type^12^,^13^,^14^. Thus, we used B95a cells, which are marmoset lymphoblastoid cells, to investigate the mechanism of persistent infection because these cells are highly susceptible to MV infection and can easily achieve 100% infection during persistent infection. In the early phase of MV infection (4 days post-infection [dpi]), most of the cells formed syncytia and died (Fig. 1a). The few remaining cells, however, started to grow again (Fig. 1a). To determine when all of the cells in culture had established a persistent infection, we monitored cellular growth and cell death during the course of MV infection (Fig. 1b and c). Although syncytium formation and cell death faded out at 2 weeks post-infection (Fig. 1a and c), cell growth was severely impaired until 28 dpi when cells started to grow in a manner similar to that of uninfected controls (Fig. 1b). A second crisis occurred between 23 and 28 dpi in all three cultures independently, prior to the re-establishment of cell growth (Fig. 1c). After the establishment of persistent infection, the cell morphology was indistinguishable from that of uninfected cells (Fig. 1a).

Staining of intracellular MV N protein confirmed that all of the cells in culture were infected with MV (Fig. 1d). Because a reduction in the amount of infectious virion release has been reported in MV persistent infection^13^, we determined the titre of released infectious virions in the persistently infected B95a cells by inoculating the supernatant onto fresh B95a cells. Although a syncytium-forming virus was released from the persistently infected B95a cells, the virus titre was two orders of magnitude lower than that obtained during acute infection (Fig. 1e). It was necessary to confirm that all of the cells in this culture were infected with MV to distinguish true persistent infection from ‘carrier culture,’ which represents a dynamic equilibrium between infected cell death and new infection of uninfected cells in culture^22^.

To more sensitively determine when during the establishment of persistent infection all of the cells had been infected, we used rMV-EGFP, a recombinant measles virus expressing enhanced green fluorescent protein (EGFP), because it allowed the direct visualisation of infected cells. Like conventional rMV, rMV-EGFP also induced a second crisis followed by the establishment of persistent infection with the same kinetics as those of conventional rMV. The majority of cells were infected with rMV-EGFP at 4 dpi, though GFP expression at 4 dpi was lower than that at 35 dpi (Fig. 1f). Until 28 dpi, we observed three populations with distinct GFP expression (GFP^negative^, GFP^low^, and GFP^high^). The expression level of GFP in GFP^low^ and GFP^high^ corresponded to that of 4 dpi and 35 dpi, respectively. GFP^negative^ cells (uninfected cells) were observed until 28 dpi, and, notably, the number of uninfected cells was increased at 21 dpi, which coincided with the time of the second crisis (Fig. 1c). Thus, the continuous infection of uninfected cells continued until 28 dpi and was particularly prevalent during the second crisis.

### Virus mutation is dispensable for persistent infection

**Figure 2:**
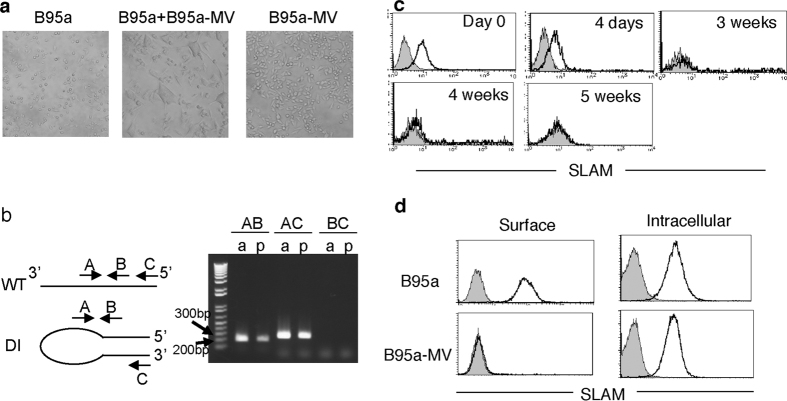
Syncytium formation when surface SLAM is downregulated. (**a**) From left to right: B95a cells, co-cultured B95a and B95a-MV cells, and B95a-MV cells. (**b**) Schematic of the MV genome (upper) and 5′ copy back DI genome (lower). Arrows indicate primers used for detection of the DI genome. The gel image shows the results of RT-PCR for the MV genome and DI genome, with acutely and persistently MV-infected B95a cells indicated by “a” and “p”, respectively. The expected length of the PCR products of primer pairs AB and AC are 206 and 266 bp, respectively. The sizes of the molecular size marker bands near the PCR products are indicated. (**c**) SLAM expression on the surface of B95a cells at the indicated time-points after MV infection. Filled and open histograms indicate uninfected and persistently MV-infected B95a cells, respectively. (**d**) Surface and intracellular SLAM staining of B95a and B95a-MV cells.

In previous studies, it was not clear whether mutations found in the viral genome were the cause or the result of long-term persistent infection because the mutations were analysed after SSPE onset or after a long period of *in vitro* culture. To address this question, we investigated if the appearance of mutant virus was involved in the establishment of persistent MV infection. First, we determined if functional H and F proteins were expressed on the surface of persistently infected B95a cells by co-culturing them with uninfected B95a cells, as it has been reported that the fusogenic activity of MV is related to its cytotoxicity^29^. Persistently infected B95a cells formed syncytia with uninfected B95a cells within 2 h (Fig. 2a). This finding indicates that persistently infected B95a cells express functional H and F proteins on the surface and that they cause cell-to-cell fusion via the virus receptor, signalling lymphocyte activation molecule (SLAM), which is highly expressed on the cell surface of uninfected B95a cells.

DI particles were shown to be involved in persistent infection with MV^19^. To further examine the involvement of DI particles in MV persistence, we aimed to assess 5′ copy-back DI particle genomes by reverse-transcription polymerase chain reaction (RT-PCR). Although it is difficult to prove the absence of DI particles, DI particles were not observed either in acute or persistent MV infection in our system (Fig. 2b). The relative expression of MV genes in persistently infected cells showed a gradient of expression like that typically seen in viruses of the order *Mononegavirales* (Fig. S1)^30^. These results indicate that aberrant gene expression and aberrant genomic fragment accumulation are not involved in persistent MV infection.

Finally, we determined the consensus genomic sequence of the persistent virus. The whole viral genome sequence was amplified by PCR as fragments and sequenced directly (data not shown). In agreement with our other results, this analysis did not reveal any mutations in persistent MV.

### Down-modulation of surface SLAM prevents syncytium formation

Because persistent MV replicated well in culture in the absence of viral mutations, without causing cell death, and successfully released infectious virus into the supernatant (Fig. 1e), we investigated if the cellular features of cells persistently infected with MV were altered. We examined the surface expression of the MV receptor SLAM in infected cells during the course of persistent MV infection. The surface expression of SLAM was gradually down-modulated beginning at 4 dpi, and it completely disappeared by 3 weeks after infection (Fig. 2c). Intracellular staining of SLAM showed that SLAM protein was expressed in the cytoplasm in persistently infected cells to a similar extent as in the parental B95a cells (Fig. 2d), indicating that the expression of this molecule at the cell surface was suppressed.

### vRNA is kept at low levels in the persistent phase of MV infection

**Figure 3:**
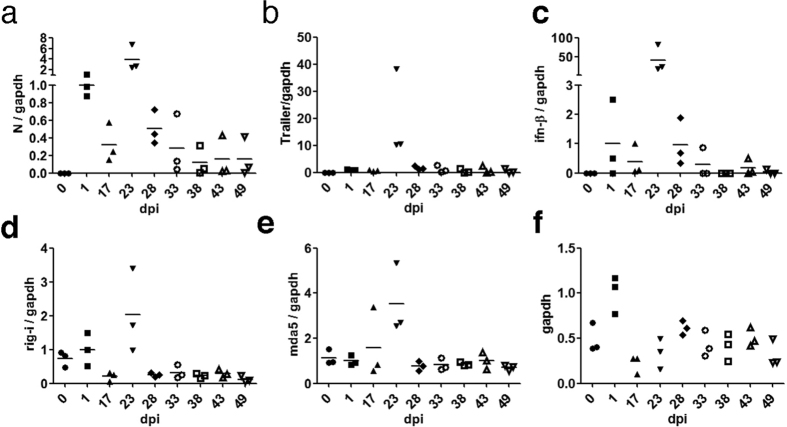
Viral RNA levels and IFN response during the establishment phase of MV persistent infection. The ratios of MV N protein (**a**), MV-Trailer (**b**), *ifnb1* (**c**), *rig-I* (**d**), and *mda5* (**e**) to *gapdh* in three independent infections are graphed. Horizontal bars indicate averages. Segmented y-axes are used in (**a**) and (**b**). (**f**) Relative amounts of *gapdh* are graphed. The cell numbers were adjusted at the time of cell harvest, except at 1 dpi because identical numbers of cells were inoculated the previous day.

To investigate the mechanism responsible for the reduction in infectious virion release during the persistent phase of MV infection, MV RNA levels were monitored during the establishment of persistent infection. The gene expressions of IFN-β and IFN response genes MDA5 and RIG-I^31^,^32^ were also examined because IFN has been suggested to be involved in the establishment of persistent MV infection. MV RNA increased after 1 day of infection and then increased again between 17 and 23 dpi, followed by a decline until 28 dpi (Fig. 3a). After this period, the vRNA level remained low. Gene expression of IFN-β, RIG-I, and MDA5 were also induced in response to vRNA accumulation (Fig. 3a). Although we were unable to accurately determine the percentage of the cells that were infected at 24 h post-infection because of the syncytium, the virus infection clearly did not reach all of the cells at this time point. Thus, the expression levels of MV N protein, IFN-β and IFN response genes that were normalised with *gapdh* expression levels at 24 h post-infection (Fig. 3b) are probably underestimated. Nevertheless, the expression levels of MV N protein and IFN-β at this time point were higher than those of cells in the persistent phase of MV infection. These data indicate that both IFN production and signalling pathways are functional in the early phase of MV infection, prior to the establishment of persistence. When the persistent infection of MV is established after 28 dpi, the expression of these genes declines to basal levels, at which their expression is maintained. Thus, IFN does not seem to be involved in the maintenance of persistent MV infection.

### Expression levels of the IFN-β gene and IFN response genes were not impaired in B95a cells persistently infected with MV

**Figure 4:**
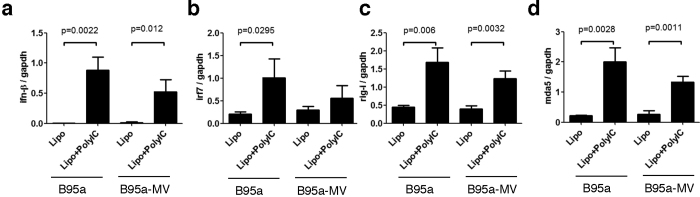
RNA sensing in persistently MV-infected B95a cells. B95a cells and persistently MV-infected B95a cells (B95a-MV) were stimulated with a complex of Poly I:C and Lipofectamine (Lipo) or with Lipo alone. Twenty-four hours later, the mRNA levels of *ifnb1* (**a**), *irf7* (**b**), *rig-I* (**c**), and *mda5* (**d**) were quantified by real-time RT-PCR. Ratios against *gapdh* are graphed. Error bars are SD (n = 3).

It has been reported that both IFN production and signalling pathways are impaired by MV^5^,^33^. To investigate if IFN production and IFN signalling pathways were impaired in cells that are persistently infected with MV, we examined the responsiveness of persistently infected B95a cells to poly I:C stimulation. Poly I:C treatment induced the gene expression of IFN-β, IRF7, RIG-I, and MDA5 in B95a cells that were persistently infected with MV as well as in uninfected control B95a cells (Fig. 4a–d). Thus, it appears that the functions of vRNA-sensing and IFN signalling pathways are not impaired in these cells.

### Prolonged MV N protein accumulation

**Figure 5:**
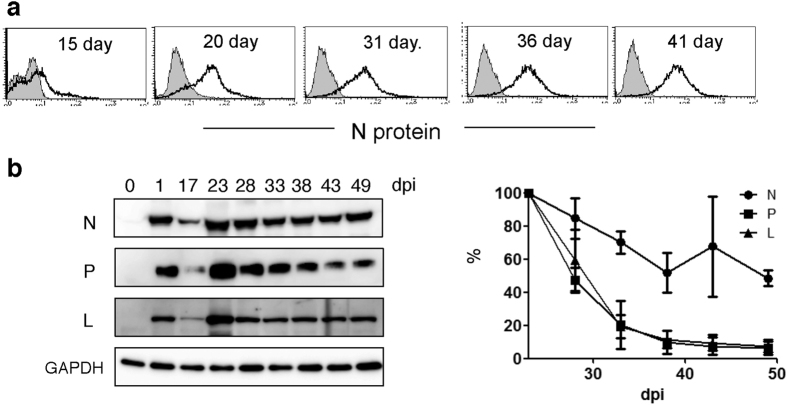
MV N, P, and L protein levels in MV-infected B95a cells. (**a**) Intracellular MV N protein was stained at the indicated dpi. Open and filled histograms indicate uninfected and infected B95a cells, respectively. (**b**) Western blotting of MV N, P, and L proteins at the indicated dpi. GAPDH was used as an internal control. Averages of the relative protein amounts of N, P and L proteins at 23 dpi in three independent infections are graphed.

Next, we aimed to determine how MV restricts its own replication during persistent infection without causing any alterations in either the viral genome sequence or the host antiviral response. We investigated this by monitoring the levels of intracellular viral proteins, and we began by using a FACS analysis to examine the intracellular N protein expression because the N protein is the most abundant viral protein. We found that the level of this protein was sustained during the establishment phase of persistent MV infection (Fig. 5a). Thus, we next focused on the accumulation of other viral proteins. The accumulation of viral proteins that are required for replication was kinetically monitored by western blotting assays. The expression levels of P and L proteins each increased at 1 and 23 days after infection and then declined quickly, following the kinetics of vRNA levels (Figs 3a and 5b). The N protein level was sustained for a longer period than the levels of P and L proteins and vRNA (Figs 3a and 5b).

### Overexpression of MV N protein interferes with MV replication

**Figure 6:**
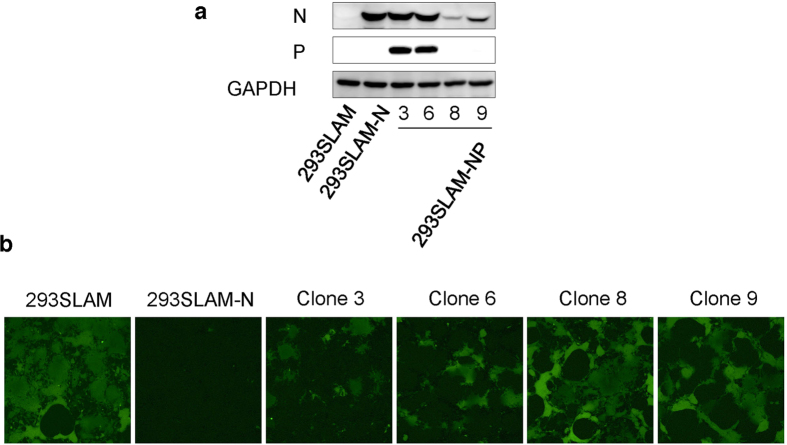
Effect of MV N overexpression on MV replication. (**a**) Expression of MV N protein and P protein in 293SLAM cells, stably MV N-expressing 293SLAM cells (293SLAM-MV-N), and 293SLAM cell clones expressing MV N and P protein with different expression levels (293SLAM-NP clones #3, 6, 8, and 9). GAPDH was used as an internal control. (**b**) 293SLAM, 293SLAM-MV-N cells, and 293SLAM-NP clones were infected with rMV-EGFP at a MOI of 0.1. Representative fluorescence microscopy images of four independent infections are shown.

Based on these observations and the fact that N protein has known a binding capacity for P protein, viral genome, and cellular factors^34^, we hypothesised that the over-accumulation of N protein competes with the viral genome for binding to the P-L complex and to the cellular factors that are required for MV replication. To examine this possibility, we established 293SLAM cells stably expressing N protein with or without different levels of P protein (293SLAM-N, 293SLAM-NP clones #3, 6, 8, and 9; Fig. 6a) and then infected these cells with rMV-EGFP to monitor viral replication. The viral replication was suppressed by the overexpression of N protein in 293SLAM-N cells, but the expression levels of N protein in 293SLAM-NP clones #8 and 9 were not sufficient to block MV replication. Although the expression levels of N protein in clones #3 and 6 were comparable to that in the 293SLAM-N cells, the suppression of MV was restored by the co-expression of P protein (Fig. 6b). Thus, the accumulation of N protein to sufficiently higher levels than that of P protein causes a downregulation of MV viral replication.

## Discussion

In this study, we presented one of the possible mechanisms by which MV establishes long-term persistent infection without mutations in the viral genome. During acute infection, MV induces IFN production and cell death. The termination of the cytopathic effect and the IFN production in the persistent phase is achieved by a combination of receptor downregulation and viral replication restriction. Although moderate amounts of surface SLAM downregulation by MV H protein have been reported^35^,^36^, we show here that long-term persistent infection with MV induces a virtual disappearance of surface SLAM. MV is able to evade the innate immune system during the persistent phase of infection by keeping vRNA levels lower than the host detection sensitivity for vRNA sensing. Low vRNA levels in the persistently infected cells also explain the observed reduction in infectious virion release, a phenomenon that was also reported by earlier studies^13^, as well as the observations in clinical cases of an absence of viremia in the late phase of MV infection and before the onset of SSPE^23^,^37^.

MV can keep its genomic RNA level constant in cells undergoing either a slow or fast cell cycle and can do so even in resting cells, such as neurons^38^. In the present study, we found that N protein functions as a stabilizer of vRNA during persistent infection. MV produced N protein during replication in persistently infected cells, and we observed that the accumulation of N protein suppressed viral replication. In our proposed model, to avoid losing the vRNA by cell division, replication could be restarted when the N protein is sufficiently diluted by cell division. This model also provides a possible explanation for the previously reported observation that cells persistently infected with MV are resistant to superinfection with a closely related virus but are not resistant to superinfection with distantly related viruses^13^. As shown in this study, pre-existing N protein suppresses the replication of superinfected MV. Thus, N protein may also prevent the replication of closely related viruses, because the N, P, and L proteins of MV are compatible among the Morbilliviruses, but they are not compatible with similar proteins from other viruses^39^,^40^.

MV replicates exclusively in the cytoplasm, so most of the viral proteins localise to this site. However, the cytoplasmic localisation of the N protein depends on the P protein because, in the absence of P protein, the N protein localises to the nucleus^41^. Therefore, if the N protein levels are in excess of the P protein levels, the excess N protein would be expected to translocate into the nucleus. Nuclear inclusion bodies composed of the N protein are indeed observed in SSPE brain specimens and in cells persistently infected with MV^41^,^42^. These observations support the over-accumulation of the N protein during persistent MV infection *in vivo*.

The type of persistent MV infection observed in the present study seems to be suitable for maintaining lifelong protective immunity against MV. In acute infection, the virus replicates vigorously and induces IFN and immunogenic cell death until the MV receptor is downregulated and N protein accumulates to a sufficient level^43^. The immune system then establishes a strong immunity against MV. During the persistent phase, the virus hides itself from immune detection by restricting viral replication. Trace amounts of viral antigen may continuously stimulate the immune system, allowing the induction of lifelong protective immunity without the clonal deletion or the cytotoxic T cell unresponsiveness induced by the presence of abundant antigen and the continuous IFN secretion that is seen in chronic infection with lymphocytic choriomeningitis virus and hepatitis C virus^44^,^45^,^46^.

## Methods

### Cells, antibodies, and viruses

B95a and 293SLAM cells were described previously^47^,^48^. Anti-human SLAM antibody (A12) was purchased from Biolegend. Alexa Fluor 488-conjugated anti-mouse and anti-rabbit IgG antibodies were purchased from Life Technologies. Anti-GAPDH mouse monoclonal antibody was purchased from Millipore. Rabbit anti-MV N protein, anti-L protein serum, and monoclonal mouse anti-MV P protein antibody were described previously^49^. Recombinant MV HL strain (rMV-HL) and rMV-EGFP were described previously^50^. Briefly, the HL strain is a wildtype virus that uses SLAM and nectin-4, but not CD46, as receptors. The rMV-EGFP was constructed by inserting an EGFP-coding sequence between the N and P genes of MV-HL cDNA. These viruses were propagated and titrated in B95a cells^49^. The pCAGzc-MV-N was constructed by inserting the coding sequence of the N protein from the HL virus strain into the EcoRI and NotI sites of the vector pCAGzc^51^. The pCAGzc-MV-N with or without pCAG-MV-P was then transfected into 293SLAM cells followed by selection with Zeocin for 293SLAM-MV-N and 293SLAM-N/P cells.

### Establishment of persistently infected cells

B95a cells were infected with the indicated viruses at a multiplicity of infection (MOI) of 0.01. Culture medium was changed every 5 days until cells started to grow without obvious syncytium formation and cell death. The cell numbers were counted, and 5 × 10^5^ cells were inoculated into new culture dishes every cell passage from 17 dpi. The initial MV infections were performed in three separate dishes, and the infected cells were then passaged independently.

### Virus genome sequence analysis

Total RNA was prepared from persistently MV-infected B95a cells. Complementary DNA was synthesised with random primers and PrimeScript reverse transcriptase (Takara Bioscience). The viral genome sequence was amplified in small segments covering whole virus genome by PCR with Phusion High-Fidelity DNA polymerase (New England Biolabs). PCR fragments were directly sequenced with BigDye Terminater v3.1 (Applied Biosystems) and 3130 Genetic Analyzer (Applied Biosystems). The resulting sequences were then compared with the original genome sequence of rMV-HL.

### RT-PCR

Total RNA was purified with Isogen (Nippongene) according to the manufacturer’s instructions. Complementary DNA was synthesized with Prime script Reverse Transcriptase (TAKARA) and random hexamers. Real-time PCR analysis was performed with THUNDERBIRD SYBR qPCR Mix (Toyobo). For analysis of the relative expression of each MV gene, cDNA was synthesised with oligo-dT primers and an MV full genome plasmid^55^ was used as a standard. For detection of 5′ copy-back DI particle genomes, primers A, B, and C were used for PCR. All of the primers used in this study are listed in Supplementary Tables S1 and S2.

### Flow cytometry

Surface SLAM was stained with anti-human SLAM antibody (A12; Biolegend) diluted 1:1000 in phosphate-buffered saline (PBS) supplemented with 2% foetal bovine serum (FBS-PBS), followed by Alexa fluor 488-conjugated anti-mouse IgG (Life Technologies) diluted 1:2000 in 2% FBS-PBS. For intracellular staining, cells were fixed and permeabilised in 70% cold ethanol on ice for 30 min. Cell were washed with PBS and stained with anti-MV N protein rabbit serum and Alexa fluor 488-conjugated anti-rabbit IgG (Life Technologies). Cells were analysed with a FACSCalibur machine (BD Bioscienses) and CellQuest software (BD Biosciences). For cell cycle analysis, cells were incubated with 0.5 μg/ml of RNase A (Sigma-Aldrich) and 40 μg/ml of propidium iodide (Sigma-Aldrich) at 37 °C for 30 min and then analysed by FACS.

### Western blot assays

Anti-GAPDH mouse monoclonal antibody was used as an internal control. A total of 1 × 10^5^ cells were directly lysed in sodium dodecyl sulfate (SDS) sample buffer, and 20 μl of the lysates were loaded into gels for SDS polyacrylamide gel electrophoresis (PAGE). MV L, N, and P proteins were separated on 8% and 10% SDS-PAGE gels and transferred onto polyvinylidene fluoride membranes (Millipore). MV N, P, and L proteins were detected with anti-MV N protein serum diluted 1:1000, anti-MV P protein antibody diluted 1:1000, and anti-L protein serum diluted 1:200, respectively, in Can Get Signal immunoreactions enhancer Solution 1 (TOYOBO), followed by incubation with an appropriate secondary antibody conjugated with HRP (DAKO) diluted 1:3000 in Can Get Signal Enhancer Solution 2. The resulting signals were developed with ECL prime (GE Healthcare) and detected with a LAS4000mini (GE Healthcare).

## Additional Information

**How to cite this article**: Doi, T. *et al*. Measles virus induces persistent infection by autoregulation of viral replication. *Sci. Rep.* **6**, 37163; doi: 10.1038/srep37163 (2016).

**Publisher's note:** Springer Nature remains neutral with regard to jurisdictional claims in published maps and institutional affiliations.

## Supplementary Material

Supplementary Figure S1
