# Supplementary material for: Measles virus induces persistent infection by autoregulation of viral replication
Source: Sci Rep. 2016 Nov 24;6:37163. doi: 10.1038/srep37163 (PMC5121633; doi:10.1038/srep37163)
Supplement: Supplementary Figure S1 [file srep37163-s1.pdf]

# Measles virus induces persistent infection by autoregulation of viral replication

**Tomomitsu Doi, Hyun-Jeong Kwon, Tomoyuki Honda, Hiroki Sato, Misako Yoneda, and Chieko Kai**

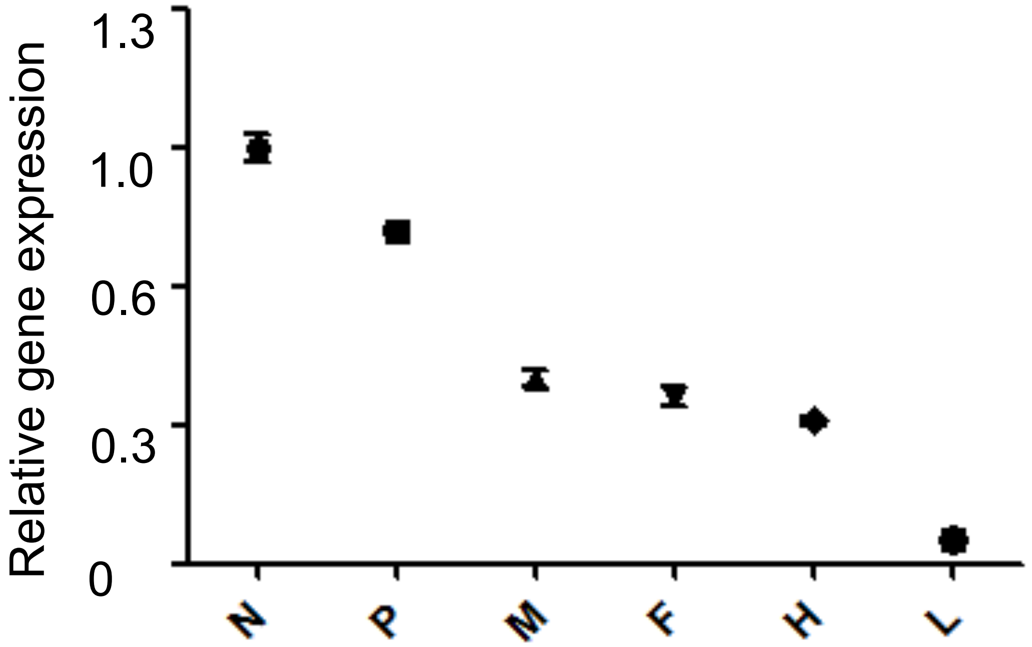


**Figure S1: Relative expression of MV genes in persistently infected cells.** Gene expression of MV genes in MV persistently infected B95a cells were measured by qRT-PCR with MV full genome plasmid as reference. Averages of triplicate samples were graphed. Error bars are SD.

Table S1. Primers used for DI particle detection

| Primer name | Seqence |
|---|---|
| A | 5′-CTTACACCAGAATATCTTCG-3′ |
| B | 5′-ATAATGCCTAACTACCTAGG-3′ |
| C | 5′-ACCAGACAAAGCTGGGAATA-3′ |

Table S2. Primers used for qPCR

| Gene | Forward primer | Reverse primer |
|---|---|---|
| gapdh | 5′-GCCTTCCGTGTCCCCACTGC-3′ | 5′-CCTCCGACGCCTGCTTCACC-3′ |
| MV-N | 5′-AACCGGGTCCAGCAGAGCAAGT-3′ | 5′-TAGGGGTGTCCGTGTCTGAGCCT-3′ |
| MV-P | 5’-ATACCGAGGGATATGCTATC-3’ | 5’-GCTTCGGAAAGTTGTTGCCTCT-3’ |
| MV-M | 5’-ATGAGCATCACCCGTCTTT-3’ | 5’-CCGATGTGGACCATAAATGT-3’ |
| MV-F | 5’-GCACAGAATTGACCTCGGTC-3’ | 5’-GATGACTCCAACAATTCCTT-3’ |
| MV-H | 5’-AGCTGTCTCAAAGGGAAACT-3’ | 5’-TTTGTCATATGGAACACCGG-3’ |
| HV-L | 5’-TCGGGTTCTATGTTGATCAC-3’ | 5’-CTCCCATTCTGTGTTCGACA-3’ |
| MV-Trailer | 5′-CTTACACCAGAATATCTTCG-3′ | 5′-ACCAGACAAAGCTGGGAATA-3′ |
| *ifn-β* | 5′-TGCTCTCCTGTTGTGCTTCT-3′ | 5′-TGCGGTTGTGTGATCTCCTT-3′ |
| *irf7* | 5′-CTGGGGGACAATCTGCTGAC-3′ | 5′-CCCTCTGCTGCCCATTTGTA-3′ |
| *rig-I* | 5′-CAGGAATGACTCTCCCAGCG-3′ | 5′-TCAGCAACTGAGGTGGCAAT-3′ |
| *mda5* | 5′-AACCAGCCTTGGAAGGGAAG-3′ | 5′-GGCAATGTAAACGGCCACTC-3′ |
